# An in-depth comparison: first wave of mpox in Rio de Janeiro, Brazil and Lisbon, Portugal

**DOI:** 10.1186/s12879-026-14087-8

**Published:** 2026-08-07

**Authors:** Raffaele Junior Aliberti, Mayara Secco Torres da Silva, Valdiléa Gonçalves Veloso, Beatriz Grinsztejn, Rosa Maria Figueiredo Teodósio, Mayumi Duarte Wakimoto

**Affiliations:** 1 Instituto de Higiene e Medicina Tropical, IHMT, Universidade NOVA de Lisboa, UNL, Rua da Junqueira 100, Lisboa, 1349-008 Portugal; 2 Hospital Santo António dos Capuchos, ULS - São José, Rua José António Serrano, Lisboa, 1150-199 Portugal; 3 Instituto Nacional de Infectologia Evandro Chagas, Fiocruz, Av. Brasil, Manguinhos, Rio de Janeiro 4365 Brazil; 4 Global Health and Tropical Medicine, GHTM, Associate Laboratory in Translation and Innovation Towards Global Health, LA-REAL, Rua da Junqueira 100, Lisboa, 1349-008 Portugal

**Keywords:** Mpox, People with HIV, Comparative epidemiology, Disease surveillance, Hospitalization, Clinical presentation, Brazil, Portugal

## Abstract

**Background:**

The 2022 mpox outbreak spread rapidly beyond endemic regions, affecting multiple countries with broadly similar transmission patterns but different public health responses and surveillance systems. We compared laboratory-confirmed mpox infections reported during the first epidemic wave in Rio de Janeiro, Brazil, and Lisbon, Portugal, with particular attention to demographic characteristics, clinical presentation, transmission patterns, hospitalization, and the role of HIV status.

**Methods:**

We conducted a retrospective analysis of routinely collected, deidentified surveillance data from national notification systems in Brazil and Portugal. Adults with laboratory-confirmed mpox reported between May and December 2022 were included. Variables were harmonized across datasets, and missing data were retained without imputation. Descriptive analyses were performed by city and HIV status. Group comparisons used Pearson’s chi-square test, Fisher’s exact test, Mann-Whitney U test, and two proportion Z tests, as appropriate. Data completeness was also explored.

**Results:**

A total of 1,614 individuals were included: 960 from Rio de Janeiro and 654 from Lisbon. Mean age was similar in both cities (34.5 vs 34.9 years), but Lisbon had a higher proportion of males (99.4% vs 93.0%). The epidemic curves showed a similar rise-and-decline pattern, with an earlier peak in Lisbon (June 2022) and a later peak in Rio de Janeiro (August 2022). Skin or mucosal lesions were the most frequently reported manifestation in both settings, affecting more than 85% of individuals. Fever was reported more often in Rio de Janeiro, whereas adenopathy and asthenia were more commonly recorded in Lisbon. Within each city, symptom profiles were not significantly different between people living with HIV and those not living with HIV. Hospitalization rates were low overall in both cities (5.7% in Rio de Janeiro and 5.4% in Lisbon), although people living with HIV in Rio de Janeiro had higher odds of hospitalization than those without HIV. Data completeness was generally better in Rio de Janeiro than in Lisbon.

**Conclusions:**

Rio de Janeiro and Lisbon experienced similarly shaped first wave mpox epidemics, despite differences in timing, surveillance completeness, and health system context. HIV status did not appear to meaningfully modify acute clinical presentation, while hospitalization risk may have been influenced by differences in immune status and care pathways. These findings highlight the importance of harmonized, high-quality surveillance to support more accurate cross-country comparisons and better targeted public health responses.

**Supplementary Information:**

The online version contains supplementary material available at https://doi.org/10.1186/s12879-026-14087-8.

## Introduction

Mpox is a zoonotic infection caused by monkeypox virus (MPXV), an Orthopoxvirus historically associated with sporadic human cases and outbreaks in Central and West Africa. Since the first recognized human infection in 1970, mpox was mainly framed as a zoonotic disease, with limited human-to-human transmission [1, 2].

This view became increasingly incomplete. After smallpox eradication and the discontinuation of routine smallpox vaccination, population immunity against orthopoxviruses declined, while mpox surveillance and research remained uneven and often under-resourced in endemic regions [3]. Signals of changing transmission were already apparent during the 2017 resurgence in Nigeria, where cases among young adults, genital lesions, HIV or other sexually transmitted infection coinfections, and probable spread through close or intimate contact networks were reported [4, 5].

The 2022 multi-country outbreak marked a major shift. Sustained transmission was documented across multiple non-endemic countries, initially in Europe and subsequently in the Americas and other regions [6]. The outbreak disproportionately affected adult men, particularly gay, bisexual, and other men who have sex with men, and transmission was strongly associated with close physical and sexual contact [7, 8]. Clinical presentation also differed from classical descriptions, with frequent localized or anogenital lesions, fewer systemic prodromal symptoms, and overlap with sexually transmitted infections [9]. These features made mpox a challenge not only for infectious disease surveillance, but also for sexual health services, community engagement, stigma reduction, and equitable access to prevention.

Portugal and Brazil were both substantially affected during the first epidemic wave, but in different health-system and surveillance contexts. Portugal detected some of the earliest cases in Europe and rapidly implemented national guidance for case detection, laboratory confirmation, contact tracing, and post-exposure vaccination [10, 11]. Brazil experienced a later epidemic peak, with Rio de Janeiro becoming one of the country’s main urban centres of transmission [12, 13]. Although both countries relied on mandatory notification and molecular confirmation, differences in surveillance systems, diagnostic pathways, data completeness, vaccination timing, and healthcare access may have shaped what was observed and recorded [11, 14].

Lisbon and Rio de Janeiro therefore provide an informative comparison of the first mpox wave in two urban settings with different population sizes, resource contexts, and public health responses. However, such a comparison requires caution.

In this study, we compared laboratory-confirmed adult mpox infections among individuals whose municipality of residence was recorded as Rio de Janeiro, Brazil, or Lisbon, Portugal, during the first epidemic wave in 2022. We focused on residence-based reported notification rates, epidemic timing, demographic characteristics, clinical manifestations, HIV status, hospitalization, and data completeness. By analysing these two urban cohorts side by side, we aimed to identify shared and divergent patterns while explicitly considering how surveillance systems and healthcare pathways may influence interpretation.

## Methods

In Brazil, data were obtained from SINAN, the national Notifiable Diseases Information System, which compiles case-based notifications and investigations for conditions under compulsory reporting. During the 2022 mpox outbreak, suspected, probable, and confirmed cases were subject to immediate notification, and laboratory results for MPXV detection were required to be reported to the Ministry of Health.

In Portugal, data were obtained through SINAVE, the National Epidemiological Surveillance System, which supports notification, epidemiological investigation, and laboratory reporting of communicable diseases. Portuguese data were accessed through a formal request submitted via TESSy, with authorization from Infarmed and the Comissão de Ética para a Investigação Clínica. Access to the Portuguese dataset was partial, limiting the availability of some variables initially requested.

### Case definitions and laboratory confirmation

The analysis included adults aged 18 years or older with laboratory-confirmed mpox in Rio de Janeiro or Lisbon during the first epidemic wave in 2022.

Operational case definitions followed the national surveillance guidance in place during the 2022 outbreak in Brazil and Portugal [11, 12]. In both countries, suspected and probable cases were based on compatible clinical manifestations, particularly acute skin or mucosal lesions, anogenital complaints, and/or epidemiological exposure in the preceding 21 days. Confirmed cases required detection of MPXV DNA by real-time PCR and/or sequencing in a biological sample.

For this analysis, cases were attributed to each setting according to the municipality of residence recorded in the national surveillance systems. Individual-level information on nationality and recent travel history was not available in the harmonized analytical dataset.

Laboratory confirmation was based on molecular detection of MPXV DNA. In Brazil, the Ministry of Health recommended RT-qPCR according to the CDC protocol, with preferred specimens including vesicular or pustular lesion material, swabs from skin or mucosal lesions, crusts, and, when appropriate, oropharyngeal/nasopharyngeal, perianal, or genital swabs [12]. In Portugal, national guidance recommended lesion exudate or vesicular/pustular fluid, oropharyngeal swabs, serum samples for complementary serological investigation, and anorectal swabs when clinically justified; positive or inconclusive samples could be referred to INSA for sequencing.

Individual-level laboratory metadata, including cycle threshold (Ct) values, delta Ct values, and assay platform information, were not available in the harmonized analytical datasets. Accordingly, laboratory confirmation was analysed as a binary surveillance classification.

Because geographic attribution was based on the municipality of residence recorded in the surveillance systems, reported case rates were calculated using the corresponding municipal population denominators. Rio de Janeiro was defined as the municipality of Rio de Janeiro, which had 6,211,223 inhabitants according to the 2022 Brazilian demographic census [15]. Lisbon was defined as the municipality of Lisbon, with an estimated 548,703 residents in 2022 according to the Instituto Nacional de Estatística [16].

### Statistical analysis

Continuous variables were summarized using means and standard deviations or medians and ranges, as appropriate. Normality of age distributions was assessed using the Shapiro–Wilk test, and age was compared between groups using the Mann–Whitney U test. Categorical variables were summarized as counts and percentages and compared using Fisher’s exact test or Pearson’s chi-square test with Yates’ continuity correction for 2*×*2 tables, as appropriate.

Temporal trends in missingness were assessed using separate univariable logistic regression models for each city and variable, with missingness coded as a binary outcome and time modelled as days since the first record in each city. All analyses were performed using R/RStudio (R 4.4.1). To explore the association between HIV status and recorded hospitalization while accounting for measured potential confounders, we fitted a multivariable logistic regression model using Firth’s penalized likelihood method. This approach was selected because hospitalization was uncommon and some covariate strata contained sparse observations. Recorded hospitalization was modelled as a binary outcome, with HIV status as the primary exposure. The exploratory model included city, age expressed per 10-year increase, sex assigned at birth, recorded MSM status categorized as MSM, non-MSM, or unknown, and epidemic time, defined as the number of months since the first symptom-onset month observed in each city. Interaction terms between HIV status and city and between city and epidemic time were included to allow the association between HIV status and hospitalization and temporal changes in hospitalization to differ between settings. Records with unknown HIV or hospitalization status were excluded from the multivariable analysis. Missing MSM status was retained as a separate category rather than imputed. A sensitivity analysis was performed after excluding records with unknown MSM status. Adjusted odds ratios (aORs) with 95% confidence intervals were reported. City and city-specific temporal terms were included to account partially for fixed and evolving differences between the surveillance and healthcare settings; however, differences in case ascertainment, reporting practices, healthcare access, and admission thresholds could not be directly measured or fully adjusted for.

The complete model output, primary city-specific contrasts, sensitivity analysis, and aggregate source data supporting Figures 1 and 3 and Table 2 are provided in the Supplementary Data.

### Variables and harmonization

The variables analysed included age, sex assigned at birth, date of symptom onset, HIV status, MSM status, reported probable route of transmission, hospitalization status, and reported clinical manifestations. Clinical manifestations were harmonized into comparable binary categories across datasets, including skin or mucosal lesions, fever, lymphadenopathy, headache, asthenia, myalgia, sore throat, chills, back pain, genital or perianal lesions, and other manifestations.

Because the Brazilian and Portuguese surveillance systems used different forms, variable structures, and response categories, all variables were recoded into a shared analytical structure before analysis. Categories that could not be mapped reliably across both datasets were not included in comparative analyses. When clinically related symptom terms differed between forms, they were grouped into broader standardized categories to improve comparability.

For the data-completeness analysis, values coded as missing or unknown, as well as categories that could not be harmonized reliably across the two surveillance systems, were classified as unavailable.

## Results

### Study population and reported case rates

A total of 1,614 adults with laboratory-confirmed mpox were included in the analysis: 960 people with municipality of residence recorded as Rio de Janeiro and 654 people with municipality of residence recorded as Lisbon. Using the corresponding 2022 municipal resident population denominators, the residence-based reported notification rate was 15.5 per 100,000 residents in Rio de Janeiro and 119.2 per 100,000 residents in Lisbon. These estimates are interpreted as residence-based reported notification rates, rather than true infection incidence, because they remain dependent on case detection, testing access, notification practices, and surveillance completeness.

### Temporal distribution

**Fig. 1:**
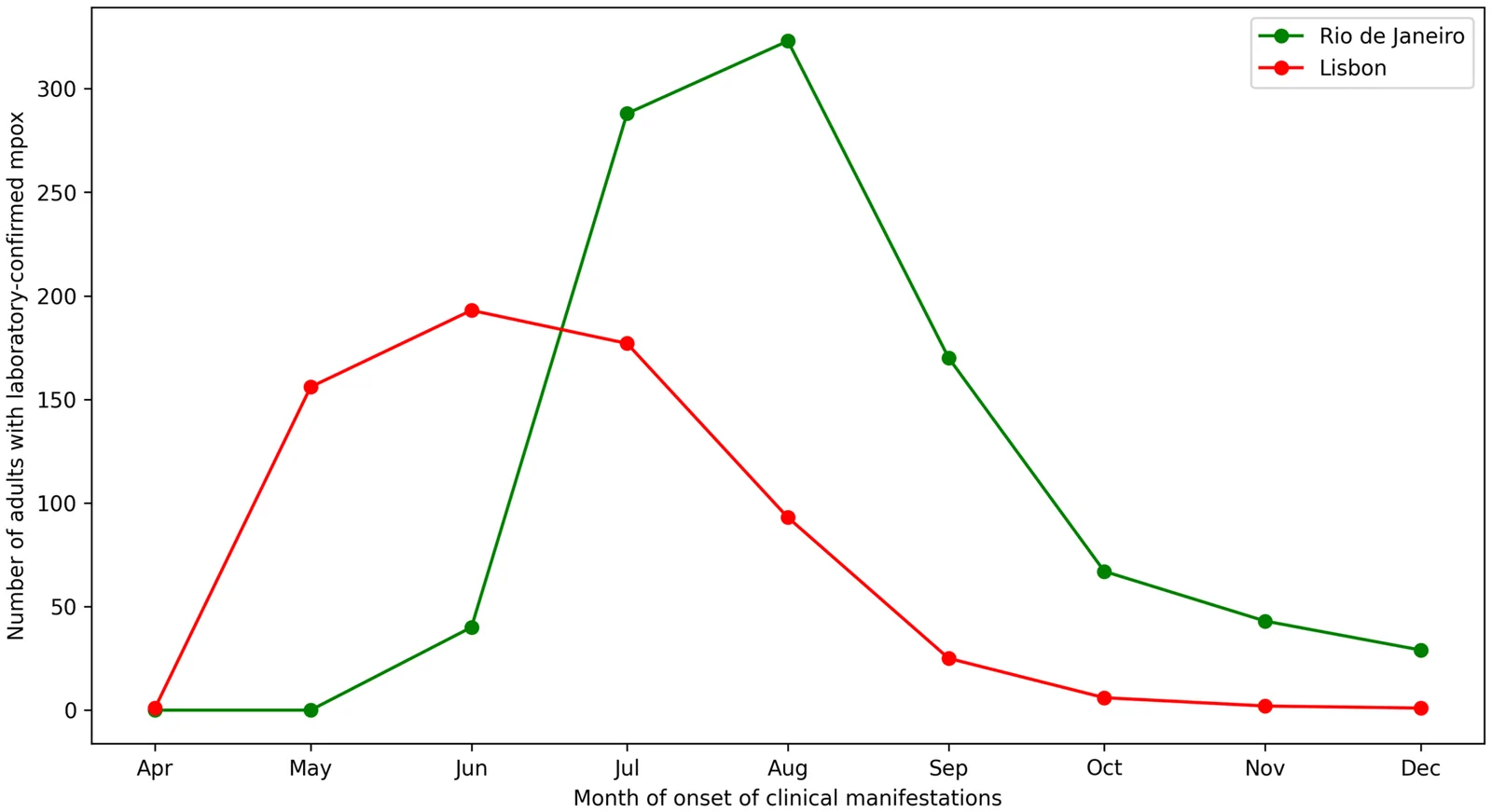
Temporal distribution of adults with laboratory-confirmed mpox by month of symptom onset in Lisbon and Rio de Janeiro, April–December 2022

Temporal analyses were based on the month of onset of clinical manifestations, which was available for all 960 individuals in Rio de Janeiro and all 654 individuals in Lisbon. In Lisbon, one case had symptom onset in April 2022, followed by a sharp increase in May and a peak in June, when 193 cases were recorded (29.5% of the Lisbon cohort). In Rio de Janeiro, the first symptom-onset records occurred in June, followed by a rapid increase in July and a peak in August, when 323 cases were recorded (33.6% of the Rio de Janeiro cohort). Case numbers subsequently declined in both settings, although the decline occurred earlier in Lisbon than in Rio de Janeiro (Fig. 1).

### Demographic characteristics

The two cohorts had similar age distributions. Mean age was 34.5 years in Rio de Janeiro and 34.9 years in Lisbon, with a median age of 33 years in both settings. Age distribution was non-normal, and the Mann–Whitney U test did not show a statistically significant difference between the two cohorts (Table 1).

**Table 1:** Demographic and epidemiological characteristics of adults with laboratory-confirmed mpox in Rio de Janeiro and Lisbon, 2022

Characteristic	Rio de Janeiro	Lisbon	Test	Result
**Overall cohort**	**n = 960**	**n = 654**		
Age (years), mean (SD)	34.49 (8.91)	34.85 (8.40)	Shapiro–Wilk	Non-normal distribution
Median (min–max)	33 (18–81)	33 (18–64)	Mann–Whitney U	*p* = 0.369
Male	893 (93.0%)	650 (99.4%)	Fisher’s exact	*p** < *0.001
Female	67 (7.0%)	4 (0.6%)		
MSM	713 (74.3%)	450 (68.8%)	Fisher’s exact	*p** < *0.001
Non-MSM	127 (13.2%)	0 (0.0%)		
Missing MSM status	120 (12.5%)	204 (31.2%)		
PLHIV	415 (43.2%)	245 (37.5%)	$\chi^2$	*p* = 0.406
People without HIV infection	488 (50.8%)	317 (48.5%)		
Missing HIV status	57 (5.9%)	92 (14.1%)		
Hospitalized	55 (5.7%)	35 (5.4%)	$\chi^2$	*p* = 0.916
Non-hospitalized	889 (92.6%)	539 (82.4%)		
Missing hospitalization status	16 (1.7%)	80 (12.2%)		
Healthcare-associated transmission	3 (0.3%)	0 (0.0%)		
Contact with contaminated material	4 (0.4%)	0 (0.0%)		
Person-to-person (non-sexual)	341 (35.5%)	0 (0.0%)		
Sexual transmission	185 (19.3%)	265 (40.5%)		
Missing route	427 (44.5%)	389 (59.5%)		
**PLHIV cohort**	**n = 415 (43.2%)**	**n = 245 (37.5%)**		
Age (years), mean (SD)	35.42 (7.95)	37.47 (8.57)	Shapiro–Wilk	Non-normal distribution
Median (min–max)	34 (19–62)	36 (22–64)	Mann–Whitney U	*p* = 0.0049
Male	413 (99.5%)	245 (100.0%)	Fisher’s exact	*p* = 0.532
Female	2 (0.5%)	0 (0.0%)		
MSM	358 (86.3%)	191 (78.0%)	Fisher’s exact	*p* = 0.011
Non-MSM	12 (2.9%)	0 (0.0%)		
Missing MSM status	45 (10.8%)	54 (22.0%)		
Hospitalized	30 (7.2%)	9 (3.7%)	Fisher’s exact	*p* = 0.120
Non-hospitalized	378 (91.1%)	212 (86.5%)		
Missing hospitalization status	7 (1.7%)	24 (9.8%)		
Contact with contaminated material	1 (0.2%)	0 (0.0%)		
Person-to-person (non-sexual)	160 (38.6%)	0 (0.0%)		
Sexual transmission	80 (19.3%)	102 (41.6%)		
Missing route	174 (41.9%)	143 (58.4%)		

*Note:* Data are presented as n (%) unless otherwise stated. SD, standard deviation; MSM, men who have sex with men; PLHIV, people living with HIV. Records with missing or unknown status were excluded from the corresponding inferential comparisons

Based on sex assigned at birth, 893 people in Rio de Janeiro (93.0%) and 650 in Lisbon (99.4%) were recorded as male, while 67 people in Rio de Janeiro (7.0%) and 4 in Lisbon (0.6%) were recorded as female.

### HIV status, MSM status and reported route of transmission

HIV status was available for 903 people in Rio de Janeiro and 562 in Lisbon. People living with HIV (PLHIV) accounted for 415 of 960 people in Rio de Janeiro (43.2%) and 245 of 654 people in Lisbon (37.5%). HIV status was unknown for 57 people in Rio de Janeiro (5.9%) and 92 in Lisbon (14.1%).

Men who have sex with men (MSM) represented the majority of people in both settings. In Rio de Janeiro, 713 people were reported as MSM (74.3%), compared with 450 in Lisbon (68.8%). Missing information on MSM status was more frequent in Lisbon than in Rio de Janeiro, affecting 204 people (31.2%) and 120 people (12.5%), respectively.

Reported routes of transmission differed between the two datasets. Sexual transmission was recorded for 185 people in Rio de Janeiro (19.3%) and 265 people in Lisbon (40.5%). Non-sexual person-to-person transmission was recorded for 341 people in Rio de Janeiro (35.5%), but was not represented as a recorded category in the Lisbon dataset. Missing information on transmission route was common in both settings, affecting 427 people in Rio de Janeiro (44.5%) and 389 people in Lisbon (59.5%).

### Clinical manifestations

**Fig. 2:**
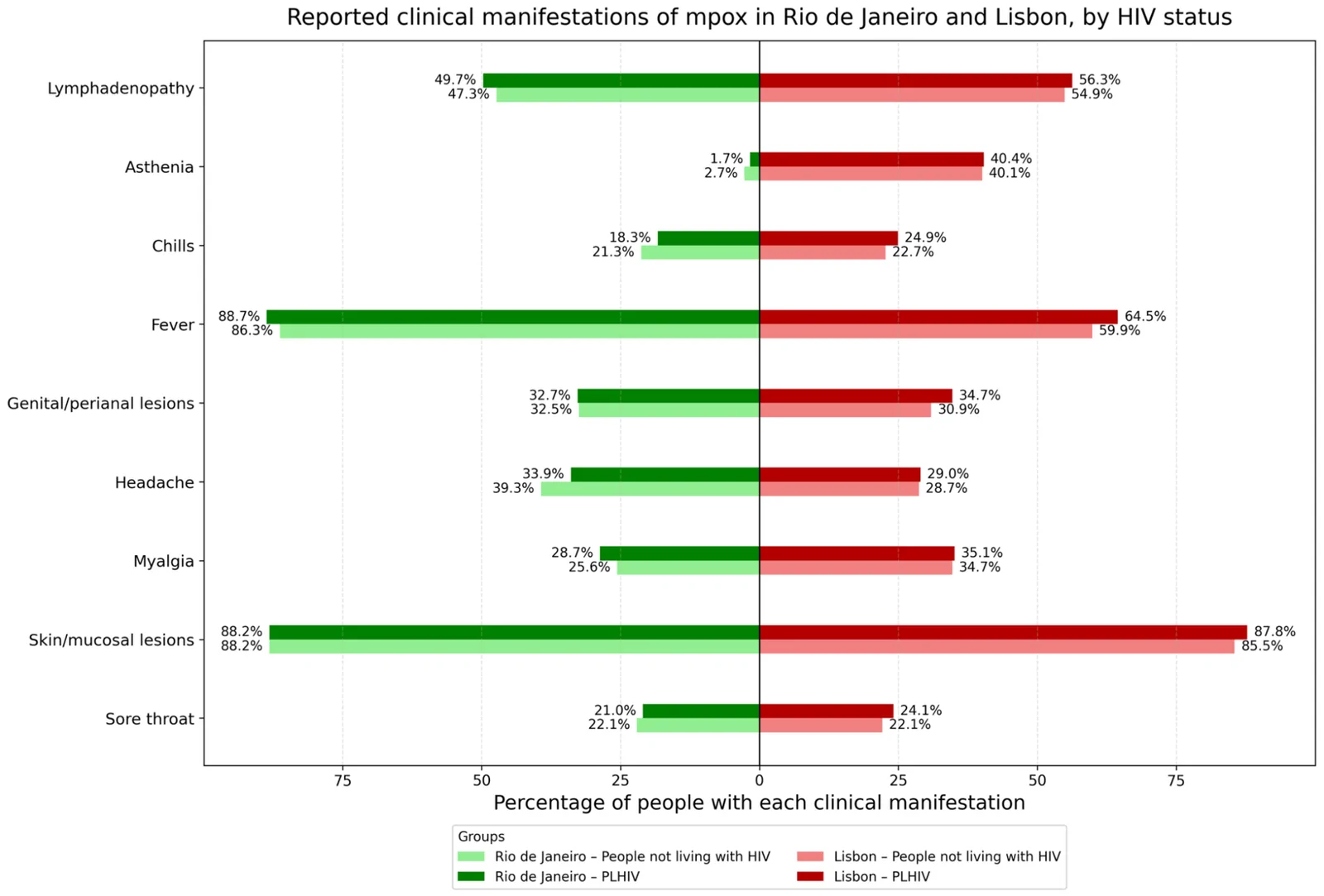
Reported clinical manifestations of mpox in Rio de Janeiro and Lisbon, stratified by HIV status. Percentages refer to the proportion of people within each city and HIV-status group with each recorded clinical manifestation. PLHIV, people living with HIV

As shown in Fig. 2, the distribution of recorded clinical manifestations was largely comparable between Rio de Janeiro and Lisbon, although some differences emerged in the relative frequency of specific symptoms. Skin or mucosal lesions were the most frequently recorded clinical manifestation in both settings, affecting more than 85% of people in each cohort. Fever was recorded more frequently in Rio de Janeiro, whereas lymphadenopathy and asthenia were recorded more frequently in Lisbon. Among systemic manifestations, fever showed the most striking between-city contrast, being recorded in 86.3–88.7% of people in Rio de Janeiro and 59.9–64.5% in Lisbon across the HIV-status strata.

When clinical manifestations were stratified by HIV status within each city, recorded profiles were broadly similar between PLHIV and people not living with HIV. No statistically significant differences in the distribution of recorded clinical manifestations were observed by HIV status within either Rio de Janeiro or Lisbon.

### Hospitalization

Hospitalization was uncommon in both settings and occurred with similar overall frequency, being reported for 55 people in Rio de Janeiro (5.7%) and 35 people in Lisbon (5.4%). However, the completeness of this variable differed between datasets: hospitalization status was missing for 16 people in Rio de Janeiro (1.7%) and 80 people in Lisbon (12.2%). Table 2 summarizes the crude and exploratory adjusted associations between HIV status and recorded hospitalization in the two settings.

**Table 2:** Crude and adjusted associations between HIV status and recorded hospitalization in Rio de Janeiro and Lisbon

Setting	PLHIV hospitalized	Not living with HIV hospitalized	Crude OR (95% CI)	Adjusted OR (95% CI)	Adjusted *p*-value
RJ	30/408 (7.4%)	21/483 (4.3%)	1.75 (0.98–3.10)	2.11 (1.14–4.05)	0.018
LIS	9/221 (4.1%)	17/301 (5.6%)	0.71 (0.31–1.62)	0.73 (0.30–1.66)	0.454

*Note:* RJ, Rio de Janeiro; LIS, Lisbon; PLHIV, people living with HIV; OR, odds ratio; CI, confidence interval. Percentages use individuals with known hospitalization status as the denominator. Adjusted estimates were obtained using Firth penalized logistic regression accounting for city, age, sex assigned at birth, recorded MSM status, epidemic timing, and the prespecified interaction terms

When stratified by HIV status, different patterns were observed between the two cities. In Rio de Janeiro, among people with available HIV and hospitalization data, hospitalization was recorded for 30 of 408 PLHIV (7.4%) and for 21 of 483 people not living with HIV (4.3%). The crude estimate suggested higher odds of recorded hospitalization among PLHIV, although the confidence interval included the null value (OR 1.75, 95% CI 0.98–3.10; Fisher’s exact *p* = 0.060). In Lisbon, hospitalization was recorded for 9 of 221 PLHIV (4.1%) and for 17 of 301 people not living with HIV (5.6%), with no clear crude association between HIV status and hospitalization (OR 0.71, 95% CI 0.31–1.62; Fisher’s exact *p* = 0.542).

The multivariable analysis included 1,413 individuals with known HIV and hospitalization status, among whom 77 had a recorded hospitalization. After adjustment for age, sex assigned at birth, recorded MSM status, epidemic timing, city, and city-specific temporal patterns, PLHIV in Rio de Janeiro had higher odds of recorded hospitalization than people not living with HIV (aOR 2.11, 95% CI 1.14–4.05; *p* = 0.018). In Lisbon, no clear adjusted association was observed (aOR 0.73, 95% CI 0.30–1.66; *p* = 0.454). The primary model suggested that the association differed between settings (ratio of odds ratios 0.34, 95% CI 0.12–0.96; *p* = 0.042). However, the Lisbon estimate and the apparent between-city heterogeneity were sensitive to the handling of missing MSM information in the sensitivity analysis. The complete model coefficients and sensitivity analysis are reported in Supplementary Tables S1 and S2.

Because detailed reasons for admission, admission thresholds, immunological markers, antiviral treatment, and disease severity indicators were not available, hospitalization was analysed as a recorded surveillance outcome.

### Data completeness

Data completeness was assessed for four key surveillance variables: MSM status, HIV status, hospitalization status, and reported route of transmission.

Overall, Lisbon showed a higher proportion of missing data than Rio de Janeiro across all four variables assessed. Missingness was particularly frequent for reported route of transmission, affecting 59.5% of records in Lisbon and 44.5% in Rio de Janeiro. Missing information was also more common in Lisbon for MSM status (31.2% vs 12.5%), HIV status (14.1% vs 5.9%), and hospitalization status (12.2% vs 1.7%).

The chronological heatmap showed that missingness was not uniformly distributed over time. In Rio de Janeiro, missing values for reported route of transmission were more frequent during the earlier phase of the outbreak and became less common over time. A similar, although less visually uniform, temporal pattern was observed in Lisbon for selected variables.

**Fig. 3:**
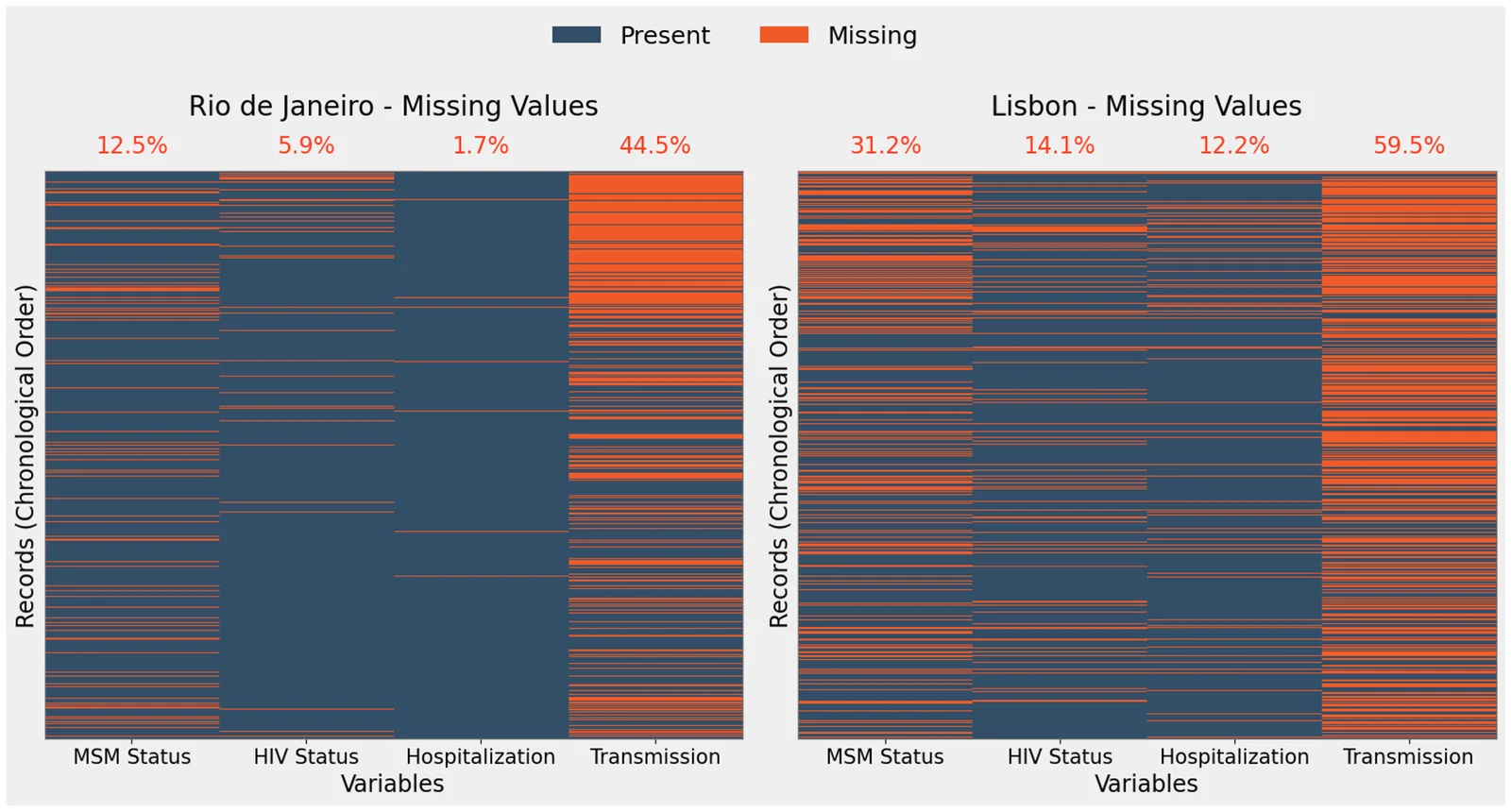
Missing data patterns for selected surveillance variables in Rio de Janeiro and Lisbon. Each row represents one record, ordered chronologically by onset of clinical manifestations. Blue indicates available information and red indicates missing information. Percentages above each column indicate the proportion of missing values for that variable within each city

Temporal trends in missingness were further assessed using univariable logistic regression models, with missingness coded as a binary outcome and time modelled as days since the first record in each city. In Rio de Janeiro, missingness decreased significantly over time for reported route of transmission (*p < 0.0001*) and HIV status (*p=0.0003*), while no significant temporal trend was observed for MSM status or hospitalization status. In Lisbon, missingness decreased significantly over time for reported route of transmission (*p < 0.0001*), MSM status (*p < 0.0001*), and hospitalization status *(p=0.037),* whereas no significant temporal trend was observed for HIV status (Fig. 3).

## Discussion

This comparative analysis of laboratory-confirmed adult mpox in Lisbon and Rio de Janeiro shows that the first epidemic wave shared several broad features across both settings, including concentration among adults, a high representation of PLHIV, predominance of skin or mucosal lesions, and relatively low recorded hospitalization. Nevertheless, the temporal distribution of cases differed between the two cities. Lisbon showed an earlier rise, with a peak in June 2022, whereas Rio de Janeiro showed a later peak in August 2022. This temporal offset is consistent with the early recognition of the 2022 mpox outbreak in Portugal, where one of the first European clusters was reported in May 2022, and the subsequent expansion of transmission in Brazil and other parts of the Americas [10, 14].

The re-emergence of mpox also needs to be interpreted within the broader historical context of declining population-level immunity to orthopoxviruses after the discontinuation of routine smallpox vaccination. Smallpox eradication remains one of the most emblematic achievements of vaccination-based public health [17]. In Portugal, the last recorded smallpox case occurred in 1952; smallpox vaccination was included in the first National Vaccination Programme launched in 1965, became non-compulsory in 1977, and ceased in 1980 following WHO-certified eradication [18]. In Brazil, the Smallpox Eradication Campaign was implemented in the 1960s and 1970s, with the disease considered eradicated nationally in 1971 and internationally certified in 1973, while vaccination activities continued in routine services until the mid-1970s [19]. Declining smallpox vaccine-induced cross-protection may have contributed to the changing epidemiology of mpox, as suggested by historical estimates of substantial protection from previous smallpox vaccination and by increased mpox incidence reported decades after mass vaccination had ceased. However, ecological, behavioural, mobility-related and surveillance factors are also likely to have contributed [3, 20].

Against this background of waning orthopoxvirus immunity, Portugal and Brazil differed in the timing and operational implementation of mpox vaccination during and after the 2022 outbreak. In Portugal, the Directorate-General of Health issued Norma 006/2022, initially recommending vaccination in a post-exposure setting for close contacts, ideally within four days and up to 14 days after exposure [21]. Portugal had received 2,700 vaccine doses donated through the European Commission/HERA, and post-exposure vaccination was available from mid-July 2022. Preventive pre-exposure vaccination for people at increased risk was introduced after the 20 September 2022 update of the DGS norm.

By 16 October 2022, 554 contacts had received post-exposure vaccination and 261 people had been vaccinated in the preventive setting, according to the Directorate-General of Health [22]. Although these data do not allow a denominator-based estimate of national vaccine coverage, they indicate early implementation and progressive uptake of the programme; by 28 July 2023, 4,823 people had been vaccinated in Portugal, corresponding to 8,009 inoculations, most of them in the pre-exposure setting [23].

In Brazil, the vaccine response was implemented later. Anvisa authorized the exceptional import and use of Jynneos/Imvanex by the Ministry of Health on 25 August 2022 [24], but national vaccination was effectively initiated only on 15 March 2023. The strategy targeted people at higher risk of severe disease, including selected PLHIV and laboratory professionals in pre-exposure settings, as well as eligible high- or medium-risk contacts in post-exposure settings [25].

In Portugal, the earlier availability of post-exposure vaccination and the subsequent introduction of pre-exposure vaccination may have contributed to the public health response during the later containment phase. Conversely, in Brazil, the delayed implementation of vaccination means that the 2022 epidemic curve was likely shaped primarily by transmission dynamics, case detection, behavioural changes, and non-vaccine public health responses rather than by vaccine-induced protection.

When focusing on sex assigned at birth, the proportion of laboratory-confirmed adult mpox diagnoses among people recorded as female at birth was higher in Rio de Janeiro than in Lisbon, accounting for 7% and 0.6% of diagnoses, respectively. This difference is consistent with Brazilian surveillance data showing that mpox diagnoses among people recorded as female at birth, although representing a minority of reported infections, became more visible later in the epidemic curve. In Rio de Janeiro State, a case series stratified by gender identity found that diagnoses among men peaked in epidemiological week 31 and then declined, whereas diagnoses among women reached their highest number in week 34, suggesting a delayed pattern that may reflect partially distinct or overlapping transmission networks. The same study reported that women diagnosed with mpox had fewer multiple sexual partnerships, less sexual contact with a potential mpox case, more frequent non-sexual contact, fewer genital lesions, fewer systemic signs or symptoms, and substantially lower HIV prevalence than men. Spatially, mpox diagnoses among men clustered in the central area of Rio de Janeiro city, while no comparable hotspot was observed among women. Although that study used gender identity rather than sex assigned at birth as its main stratification variable, its findings support the interpretation that transmission patterns among people outside the predominant male sexual networks may have been more heterogeneous and temporally delayed [26].

The distribution of recorded clinical manifestations was broadly similar between PLHIV and people not living with HIV within each city. This finding is consistent with reports from the 2022 outbreak showing that, among PLHIV with preserved immune status and access to antiretroviral therapy, mpox presentation and short-term outcomes were often comparable to those observed in people not living with HIV [8, 27]. In the large multinational case series by Thornhill et al., a substantial proportion of people with mpox were living with HIV, but most had well-controlled infection and did not show a clearly distinct clinical profile compared with people not living with HIV [8]. Similarly, a UK analysis found no major differences in mpox severity, hospitalization, or illness duration according to HIV status [27].

However, HIV status alone is an incomplete marker of clinical vulnerability. Evidence from larger cohorts and global case series indicates that severe mpox is concentrated particularly among people with advanced or uncontrolled HIV infection, especially those with low CD4 counts or unsuppressed HIV viral load [28, 29]. Fulminant, necrotizing, and disseminated forms of mpox have been described mainly in people with advanced immunosuppression, supporting the interpretation that immune status, rather than HIV serostatus alone, is the key modifier of disease severity [29].

Recorded hospitalization was uncommon in both settings. In the exploratory adjusted analysis, PLHIV had higher odds of recorded hospitalization in Rio de Janeiro, whereas no clear adjusted association was observed in Lisbon. This finding is consistent with the broader literature suggesting that hospitalization among people with mpox is not determined by HIV status alone, but is strongly influenced by immune status, comorbidities, clinical complications, and care pathways [30, 31]. The association between HIV status and recorded hospitalization in Rio de Janeiro should be interpreted cautiously. Evidence from international and Brazilian cohorts indicates that severe mpox outcomes are concentrated particularly among people with advanced or uncontrolled HIV infection, especially those with low CD4 counts or unsuppressed HIV viral load [29, 31, 32]. In the Brazilian context, hospitalizations have been associated with proctitis, pain control, secondary infections, and advanced immunosuppression, with worse outcomes clustering among people with very low CD4 counts [31]. By contrast, reports from Lisbon and other European settings have described mostly mild disease among PLHIV with preserved immune function and high antiretroviral therapy coverage, with no clear excess of severe outcomes attributable to HIV status alone [27, 33].

In the crude analysis, the estimate for Rio de Janeiro suggested higher odds of recorded hospitalization among PLHIV, although the confidence interval included the null value. In the exploratory multivariable analysis, the association remained apparent after adjustment for age, sex assigned at birth, recorded MSM status, epidemic timing, city, and city-specific temporal patterns. No clear adjusted association was observed in Lisbon. However, the Lisbon estimate and the apparent heterogeneity between cities were sensitive to the handling of missing MSM information.

These adjusted findings should not be interpreted as demonstrating an independent or causal effect of HIV status. City and epidemic timing could account only partially for differences in case ascertainment, surveillance practices, healthcare access, clinical pathways, and admission thresholds. Moreover, the datasets did not include CD4 counts, HIV viral load, antiretroviral treatment status, comorbidities, standardized measures of mpox severity, or reasons for admission. Residual and unmeasured confounding therefore remains likely, and the multivariable estimates should be regarded as exploratory adjusted associations.

Data completeness emerged as a central finding of this comparison, rather than a purely technical limitation. Lisbon showed higher missingness than Rio de Janeiro for all four variables included in the missing-data analysis, particularly reported route of transmission, MSM status, HIV status, and hospitalization status. In both settings, missingness was especially frequent for reported route of transmission, highlighting the difficulty of collecting sensitive behavioural and exposure-related information during a rapidly evolving outbreak. This is consistent with broader evidence that infectious disease surveillance systems vary in completeness, timeliness, and accuracy across settings, and that passive or routine reporting systems are particularly vulnerable to underreporting, delays, limited standardization, and differences in local data-collection capacity [34, 35].

In the context of mpox, these limitations are particularly relevant because key epidemiological variables depend not only on clinical assessment, but also on the structure of notification forms, the availability of standardized response categories, the way questions are asked, perceived stigma, trust in healthcare services, and the opportunity to disclose sexual practices and exposure histories. Evidence from the EQUALITY study showed that sexual orientation and gender identity data are more acceptable when collected through standardized, non-verbal self-report approaches, with sexual and gender minority patients reporting greater comfort and improved communication compared with verbal collection during the clinical encounter. This supports the interpretation that missingness in variables related to sexuality, gender, and transmission route may reflect the conditions under which information is collected, rather than absence of exposure or lack of epidemiological relevance [36, 37].

The chronological missing-data heatmap and logistic regression analyses further suggested that missingness was not randomly distributed. In Rio de Janeiro, missing data for reported route of transmission were more concentrated in the early phase of the outbreak and decreased over time, probably reflecting progressive adaptation of surveillance forms, reporting workflows, and professional familiarity with the new disease. In Lisbon, missingness also decreased over time for selected variables, but remained substantial for transmission-related information. These patterns indicate that differences in recorded MSM status and transmission route may partly reflect surveillance architecture, data collection procedures, coding practices, and temporal changes in reporting, rather than true differences in exposure patterns alone. Similar concerns have been described in Brazilian mpox surveillance, where information on sexual behaviour was frequently incomplete in some groups, underscoring how gender norms, stigma, and assumptions about sexual risk can shape what is asked, disclosed, and ultimately recorded [37, 38].

For cross-country mpox comparisons, this has important implications. Variables such as sexual orientation, gender identity, sex assigned at birth, reported sex between men, HIV status, and route of transmission are essential for understanding outbreak dynamics and targeting public health interventions, but they require standardized, privacy-preserving, and community-sensitive collection methods. The absence or inconsistent recording of these variables can obscure transmission patterns, reduce comparability between settings, and limit the design of equitable prevention strategies. Future surveillance systems should therefore prioritize harmonized core variables, clear operational definitions, integration into electronic reporting systems, and training for healthcare professionals in the collection of sensitive epidemiological information. In this sense, missing data should be interpreted not only as a methodological constraint, but also as an indicator of how surveillance systems performed under outbreak conditions.

## Conclusions

This comparative analysis shows that differences between Lisbon and Rio de Janeiro cannot be interpreted only as differences in epidemiology, but also as the result of distinct public health systems, surveillance structures, clinical pathways, and social contexts. In both settings, the first mpox epidemic wave exposed the importance—and the fragility—of collecting high-quality data on sensitive variables such as route of transmission, sexual practices, MSM status, HIV status, sex assigned at birth, and gender identity.

For infections linked to sexual contact or socially sensitive behaviours, missing data are not neutral. They may reflect stigma, taboo, discomfort, lack of standardized tools, or unequal opportunities for disclosure within healthcare encounters. Importantly, these mechanisms can operate across different countries in similar ways, shaping what is asked, what is disclosed, and what ultimately becomes visible in surveillance systems.

Our findings therefore underline the need to evaluate outbreak responses as complex public health processes, rather than as simple case counts. Future mpox surveillance should prioritize harmonized core variables, privacy-preserving data collection, and training for healthcare professionals in the collection of sensitive epidemiological information. Strengthening these dimensions is essential to improve comparability between settings, reduce stigma-driven blind spots, and support more equitable and community-sensitive responses to future outbreaks.

## Electronic supplementary material

Below is the link to the electronic supplementary material.

Supplementary Material 1: Complete output of the Firth penalized logistic regression model, primary contrasts, sensitivity analysis, aggregate source data supporting the figures and tables, and analytical variable coding.

## Data Availability

The datasets analyzed during the current study are not publicly available. Because off the low frequency of some variables and the potential risk of indirect identification, the database cannot be publicly shared. Access to the original surveillance data is subject to institutional, ethical, and legal restrictions imposed by the relevant national authorities in Brazil and Portugal.
